# Neurovascular Dysfunction and Glymphatic Impairment: An Unexplored Therapeutic Frontier in Neurodegeneration

**DOI:** 10.3390/ijms262411843

**Published:** 2025-12-08

**Authors:** Ghaith K. Mansour, Olena Bolgova, Ahmad W. Hajjar, Volodymyr Mavrych

**Affiliations:** 1 College of Pharmacy, Alfaisal University, Riyadh 11533, Saudi Arabia; gkmansour@alfaisal.edu; 2 College of Medicine, Alfaisal University, Riyadh 11533, Saudi Arabia; obolgova@alfaisal.edu (O.B.); awhajjar@alfaisal.edu (A.W.H.)

**Keywords:** neurovascular dysfunction, glymphatic system, blood–brain barrier, neurodegeneration, precision medicine

## Abstract

Neurodegenerative diseases pose major clinical challenges partly due to the underappreciation of the brain’s vascular and clearance systems. Evidence suggests that neurovascular dysfunction and glymphatic impairment are early contributors to disease onset, preceding established markers such as protein aggregation. This review synthesizes recent advances in understanding how disruption of the neurovascular unit (NVU) and glymphatic pathways contributes to neurodegeneration. We analyzed published literature documenting the temporal relationship between vascular dysfunction, glymphatic clearance impairment, and subsequent neurodegenerative pathology, with a focus on identifying therapeutic targets within this axis. Current research demonstrates that blood-brain barrier BBB breakdown, pericyte dysfunction, and compromised cerebral perfusion precede protein aggregation in multiple neurodegenerative disorders. Glymphatic dysfunction, characterized by aquaporin-4 (AQP4) depolarization and abnormalities in meningeal lymphatic vessels, impairs the clearance of neurotoxic metabolites. Novel therapeutic opportunities include the preservation of pericyte function, restoration of AQP4 polarity, enhancement of meningeal lymphatic drainage via vascular endothelial growth factor-C (VEGF-C)/vascular endothelial growth factor receptor-3 VEGFR-3 signaling, and targeted modulation of microRNA and complement pathways that regulate neuroinflammation. By targeting the earliest vascular and glymphatic disruptions, emerging therapeutic strategies may halt or delay disease progression before irreversible neuronal loss occurs. This neurovascular-glymphatic approach represents an unexplored frontier that complements traditional protein-centric therapeutic paradigms, offering new possibilities for early intervention in neurodegenerative disorders.

## 1. Introduction

The conventional understanding of neurodegenerative disorders has predominantly focused on protein aggregation, neuronal death, and synaptic dysfunction as primary pathogenic mechanisms [1]. However, emerging evidence reveals a critical and underappreciated pathophysiological axis that precedes and potentially drives these classic hallmarks: the neurovascular-glymphatic dysfunction cascade [2]. This review presents a comprehensive analysis of an unexplored therapeutic frontier centered on the intricate relationship between cerebrovascular integrity, glymphatic clearance mechanisms, and the inflammatory cascade that culminates in neurodegeneration.

The blood-brain barrier (BBB), once considered a static protective barrier, is now recognized as a dynamic interface critically involved in the pathogenesis of multiple neurodegenerative conditions [3]. The earliest indicators of multiple neurodegenerative disorders in humans and animal models include impaired BBB stability, regional cerebral blood flow shortfalls, and vascular inflammation associated with BBB dysfunction [4]. Concurrently, the recently discovered glymphatic system represents a fundamental brain waste clearance mechanism whose dysfunction may precede classical pathological changes in Alzheimer’s disease and other neurodegenerative disorders [2]. The convergence of BBB dysfunction, glymphatic impairment, and neuroinflammation creates a self-perpetuating cycle that accelerates neurodegeneration through mechanisms that remain largely untargeted by current therapeutic approaches. This neurovascular dysfunction represents one of the earliest detectable changes in neurodegeneration, often preceding classical pathological markers by years or decades [5].

This review identifies three critical knowledge gaps that represent unprecedented therapeutic opportunities: (1) the role of pericyte dysfunction as a primary initiator of neurovascular NVU failure, (2) the therapeutic potential of targeting glymphatic-lymphatic interfaces, and (3) the development of precision medicine approaches that address the vascular-inflammatory axis in neurodegeneration. These interconnected pathways offer novel pharmacological targets that could potentially halt or reverse the neurodegenerative process before irreversible neuronal damage occurs.

## 2. Methodology of Literature Search

A comprehensive literature search was conducted to synthesize information for this narrative review, focusing on the intersection of neurovascular, glymphatic, and neuroinflammatory pathways in neurodegeneration. The search was performed using the PubMed, Scopus, and Web of Science databases.

Our search strategy employed a combination of keywords and MeSH (Medical Subject Headings) terms. The primary search terms included: (“neurovascular unit” OR “neurovascular dysfunction” OR “blood-brain barrier” OR “pericyte”) AND (“glymphatic system” OR “glymphatic impairment” OR “meningeal lymphatics” OR “aquaporin-4”) AND (“neurodegeneration” OR “Alzheimer’s disease” OR “neuroinflammation” OR “proteinopathy”).

This primary search was supplemented with more specific secondary searches to identify key mechanisms and therapeutic targets discussed in the review. These included combinations of the above terms with “complement system”, “C1q”, “microglia”, “synaptic pruning”, “VEGF-C”, “microRNA”, “miR-124”, “miR-155”, and “sPDGFRβ”.

The search was primarily focused on articles published between January 2015 and October 2025 to ensure the inclusion of the most recent advances in this rapidly evolving field. This timeframe was expanded to include highly cited, foundational papers published prior to 2015 that were essential for establishing key concepts (e.g., the discovery of the glymphatic system or the role of the complement in synaptic pruning).

Inclusion criteria for articles were: (1) original research articles (preclinical and clinical studies), (2) review articles, and (3) meta-analyses. All articles were required to be published in the English language. The selection process involved screening titles and abstracts for their direct relevance to the review’s central themes. Full texts of the selected articles were then assessed for their contribution to the understanding of neurovascular-glymphatic interactions, key biomarkers, and emerging therapeutic targets.

## 3. Pathophysiology of Neurovascular Unit Dysfunction

### 3.1. From Components to Pericyte-Driven Pathology

**Figure 1:**
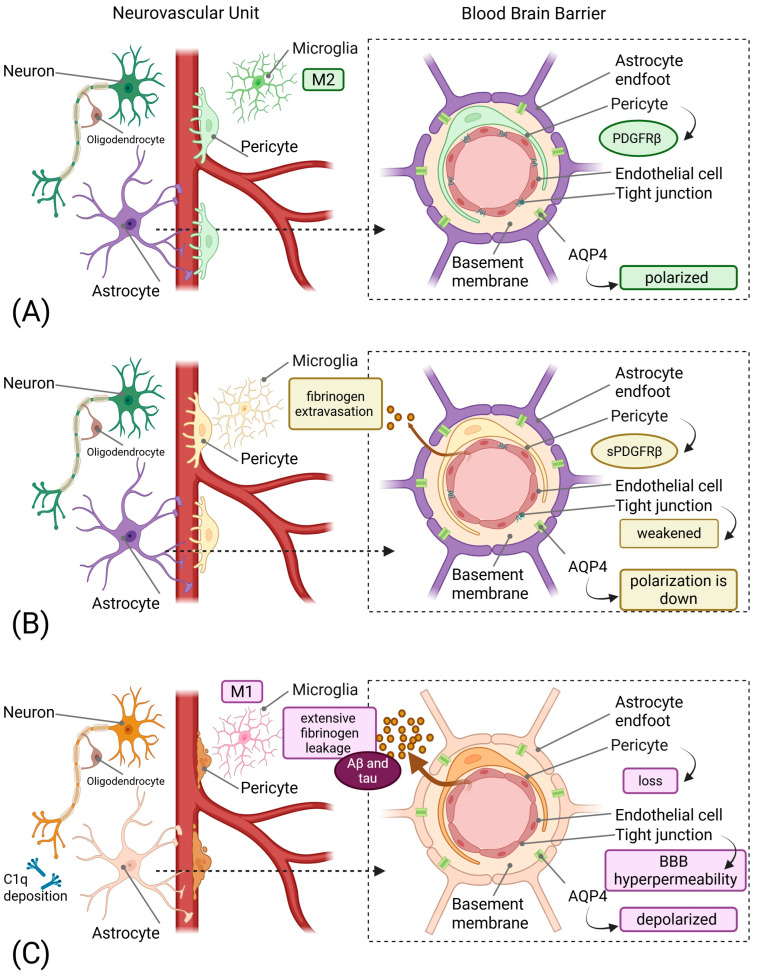
The Neurovascular-Glymphatic Dysfunction Cascade in Neurodegeneration. (**A**) Healthy NVU showing intact BBB with functional pericytes expressing PDGFRβ, endothelial cells with tight junctions, astrocytic endfeet with polarized AQP4, and resting M2 microglia. Normal cerebral blood flow and efficient glymphatic clearance are maintained. (**B**) 5–10 years before the clinical signs: Early neurovascular dysfunction characterized by pericyte injury with soluble PDGFRβ (sPDGFRβ) release, initial BBB breakdown indicated by weakened tight junctions and fibrinogen extravasation and beginning loss of AQP4 polarization. Early microglial activation is evident. These changes occur years before clinical symptoms. (**C**) Clinical symptom onset: Progressive pathology showing significant pericyte loss, BBB hyperpermeability with extensive fibrinogen leakage, impaired glymphatic clearance with protein accumulation (Aβ and tau), activated M1 microglia, complement component C1q deposition on synapses, and reactive astrocytes. Created in BioRender. Mavrych, V. (2025) https://BioRender.com/e97uydy.

The NVU comprises endothelial cells, pericytes, astrocytes, microglia, and neurons, collectively maintaining cerebrovascular homeostasis and BBB integrity [6]. The persistent neurovascular unit dysfunction (NVUD) hypothesis proposes that continuous abnormalities in the NVU following initial insults serve as the pathophysiological substrate yielding chronic neuroinflammation, proteinopathies, and oxidative stress [7]. Figure 1 illustrates the progressive cascade of neurovascular dysfunction, from early pericyte injury and subtle BBB disruption to advanced neurodegeneration characterized by severe vascular damage, protein accumulation, and chronic neuroinflammation, highlighting how these changes precede and potentially drive classical disease manifestations.

This framework suggests that targeting NVUD could provide both treatment and prevention strategies for late-onset neurodegenerative diseases, representing a paradigm shift from protein-centric to vascular-centric therapeutic approaches. High metabolic demands and continuous exposure to systemic inflammatory mediators make the NVU vulnerable [8], establishing it as a critical target for early therapeutic intervention [5]. Recent evidence demonstrates that NVUD with BBB hyperpermeability contributes to major depressive disorder and various neurological conditions through oxidative stress and neuroinflammation mechanistically linked to neurovascular dysfunction [9].

Understanding the contribution of neurovascular dysfunction with BBB hyperpermeability to neurodegeneration pathophysiology may help identify novel therapeutic and preventative approaches [10]. The temporal relationship where BBB dysfunction and decreased cerebral blood flow are early pathophysiological changes in neurodegenerative disorders suggests that vascular-targeted therapies could potentially halt disease progression before irreversible neuronal damage occurs [11]. Table 1 summarizes the key biomarkers of neurovascular and glymphatic dysfunction that can be detected in cerebrospinal fluid (CSF), plasma, and brain tissue, providing critical diagnostic and monitoring tools for both clinical assessment and therapeutic development.

Within this integrated neurovascular framework, pericytes emerge as the critical cellular component whose dysfunction initiates the cascade leading to widespread NVU failure. Pericytes are contractile cells embedded within the capillary basement membrane that have emerged as central regulators of BBB integrity and cerebral blood flow [32]. Pericyte dysfunction, characterized by the release of soluble platelet-derived growth factor receptor-β (sPDGFRβ), serves as both a biomarker of BBB dysfunction and a potential therapeutic target [15]. The loss of pericytes has been associated with the development and progression of various diseases, such as diabetes, Alzheimer’s disease, stroke and traumatic brain injury [5,33].

CSF PDGFRβ levels increase in early neurodegenerative disorders [12,14,15]. These elevations correlate with both neuroinflammation and cognitive decline [13,34]. BBB alterations may contribute to Alzheimer’s disease pathology through various mechanisms, including impaired amyloid-β clearance and neuroinflammation, with soluble PDGFRβ emerging as a potential biomarker for BBB integrity [34].

The PDGF-BB/PDGFRβ signaling pathway maintains pericyte survival and vascular stability through activation of extracellular signal-regulated kinase ERK and phosphatidylinositol 3-kinase PI3K pathways [35]. Disruption of this signaling cascade leads to pericyte loss, BBB breakdown, and subsequent neuroinflammation [36]. Notably, pericyte dysfunction appears to be particularly pronounced in APOE4 carriers, where impaired APOE-mediated signaling accelerates pericyte injury and vascular regression. APOE4 promotes the cyclophilin A-nuclear factor B-matrix metalloproteinase 9 complex pathway, which directly increases pericyte injury and impairs the formation of basement membranes [37].

Pericyte loss is one of the earliest characteristics of cerebral amyloid angiopathy, and although pericyte loss correlates with neuronal loss, the molecular mechanisms by which pericyte loss contributes to neurodegeneration remain poorly understood. BBB disruption resulting from pericyte loss serves as an early pathological hallmark in cerebral amyloid angiopathy, promoting amyloid-β accumulation and neurodegeneration via MAPK-dependent pathways [38].

### 3.2. Vascular Endothelial Growth Factor as a Dual-Acting Therapeutic Target

Vascular endothelial growth factor (VEGF) represents a critical mediator of neurovascular coupling and brain clearance mechanisms with established neuroprotective properties. VEGF prevents neurons from death under critical conditions such as hypoxia and glucose deprivation through binding to specific receptors, which are also expressed on the surface of neuronal cells. The neuroprotective actions occur directly through the inhibition of programmed cell death or apoptosis and the stimulation of neurogenesis [39]. VEGF binding to VEGFR-2 receptors triggers the phosphatidylinositol 3-kinase/Akt signal transduction system and, in consequence, leads to the inhibition of programmed cell death by activating antiapoptotic proteins through the transcription factor NF-κB and inhibiting proapoptotic signaling [39]. Recent clinical evidence demonstrates that transcranial radiofrequency wave treatment increases VEGF levels in Alzheimer’s disease patients, correlating with enhanced clearance of tau and amyloid-β proteins from the brain through facilitation of meningeal lymphatic vessel flow and toxin clearance [40].

Exogenous application of VEGF can increase the permeability of the BBB without causing brain edema, and pretreatment with VEGF may be a feasible method to facilitate drug delivery into the CNS [41]. VEGF treatment at optimal concentrations significantly reduced brain weight loss and gross brain injury in neonatal hypoxic–ischemic brain injury models. The neuroprotective effects may be related to activation of the Akt/ERK signaling pathway, as VEGF increased phosphorylation of protein kinase B and extracellular-signal regulated kinase 1/2 in the cortex [42]. The temporal aspects of VEGF treatment are critical, as early inhibition of VEGF may have significant potential against cerebral ischemia, partly by regulating the expression of matrix metalloproteinases [43]. While pericyte dysfunction initiates neurovascular unit breakdown [32,35], the consequences extend beyond simple vascular permeability to fundamentally alter brain waste clearance mechanisms [44,45].

### 3.3. The Glymphatic-Lymphatic Interface

The glymphatic system’s role in human neurodegeneration remains contentious. While rodent studies consistently demonstrate AQP4-dependent clearance mechanisms, human evidence is more limited and conflicting. Despite growing interest in neurovascular-glymphatic dysfunction, several controversies persist in the field. The existence and functionality of the glymphatic system in humans remains debated, with some studies questioning whether findings from rodent models accurately translate to human pathophysiology [4]. Smith et al. demonstrated diffusive and AQP4-independent solute transport in rodent brain parenchyma, challenging the traditional glymphatic model [22]. Additionally, the temporal relationship between vascular dysfunction and protein aggregation varies across studies, with ongoing debate about whether vascular pathology is a cause or consequence of neurodegeneration. These knowledge gaps highlight the need for human-specific research and standardized methodologies to validate therapeutic targets identified in preclinical models.

The glymphatic system, a brain-wide network facilitating CSF-interstitial fluid exchange, represents a fundamental mechanism for clearing metabolic waste and pathological proteins [44]. This system functions through perivascular pathways, where AQP4 water channels on astrocytic endfeet facilitate fluid movement [45]. Dysfunction of this system has emerged as an early and predictive marker of neurodegeneration, often preceding amyloid pathology [2]. The glymphatic system was identified as a waste drainage system in the brain that promotes the elimination of amyloid-β and tau protein [46]. Regional variation in glymphatic function dictates tau accumulation in mouse models of Alzheimer’s disease tauopathy, with impaired CSF-interstitial fluid exchange and AQP4 polarization observed in affected regions [47]. The central role of AQP4 in the glymphatic clearance of tau from the brain has been established through studies showing marked impaired glymphatic CSF-interstitial fluid exchange and tau protein clearance using novel AQP4 inhibitors [47].

Impaired glymphatic clearance is an important cause of metabolite accumulation in Alzheimer’s disease, as the disease is characterized by the abnormal accumulation of amyloid-β protein creating neuritic plaques and hyperphosphorylated tau protein forming neurofibrillary tangles [48]. Multisensory gamma stimulation has been shown to promote glymphatic clearance, as glymphatic transport clears parenchymal metabolites, including pathogenic proteins such as amyloid-β [49]. The glymphatic system, critically dependent on astrocytic AQP4 water channels for CSF-interstitial fluid exchange [21,23], becomes compromised when neurovascular integrity fails [2,47].

### 3.4. Aquaporin-4 Polarity Loss: A Therapeutic Target

The polarized localization of AQP4 at perivascular astrocytic endfeet is essential for efficient glymphatic function [45]. In Alzheimer’s disease Loss of AQP4 polarity occurs when AQP4 expression is mislocalized within astrocytes, becoming broadly distributed rather than concentrated at the perivascular end feet, impairing its efficiency in fluid transport and waste clearance, which exacerbates the accumulation of amyloid-β, contributing to the progression of Alzheimer’s disease pathology. Studies have shown that various factors, such as APOE4 and amyloid-β, influence the structure and function of AQP4, thereby regulating glymphatic system flow and affecting cognitive function. AQP4 holds great potential as a therapeutic target for Alzheimer’s disease, with drug development and lifestyle interventions, such as aerobic exercise and dietary regulation, being promising approaches to restore AQP4 polarity and enhance its metabolic waste (i.e., β-amyloid) clearance capacity [50,51].

Recent research identifies calmodulin-dependent phosphorylation of AQP4 as leading to increased expression of AQP4 at the plasma membrane of astrocytes in hypoxia-induced edema. The mechanism involves transient receptor potential vanilloid type 4-facilitated calcium influx that activates calmodulin, leading to cAMP-dependent protein kinase A activation. The phosphorylation of AQP4 at Ser276 causes AQP4 to relocalize to the plasma membrane, and inhibition of calmodulin with trifluoperazine significantly reduced AQP4 translocation, CNS edema, and accelerated functional recovery compared with untreated animals [52]. Alterations in AQP4 expression and polarization occur in neurodegenerative diseases, with depolarized AQP4 expression observed to occur in line with disease progression. AQP4 depolarization may be a pathological factor associated with disease onset and progression, as sustained depolarization of AQP4 impairs the function of maintaining water balance in the spinal cord, leading to swelling and malformation of astrocytes and interfering with neuronal function [53].

The astrocyte AQP4 polarized distribution-mediated glymphatic system is essential for amyloid-β and abnormal tau clearance and represents a potential therapeutic target for Alzheimer’s disease. High-intensity interval training has been shown to ameliorate Alzheimer’s disease pathology through enhancement of the glymphatic system via restoration of AQP4 polarization [54]. Aerobic exercise improves clearance of amyloid-β via the glymphatic system, as previous studies have suggested that aquaporin-4-mediated glymphatic system is an important pathway to clear β-amyloid in the brain [50].

### 3.5. Meningeal Lymphatic Vessels: A Novel Drainage Target

Effective glymphatic clearance requires not only proper AQP4 function but also efficient downstream drainage pathways [44,45]. The recent discovery of meningeal lymphatic vessels has revealed a critical peripheral component of brain waste clearance that directly connects central nervous system drainage to systemic lymphatic circulation [55]. These vessels, which drain approximately 50% of CSF volume, represent a direct connection between CNS and peripheral lymphatic circulation [56]. Dysfunction of meningeal lymphatic vessels (mLVs) has been implicated in protein accumulation and cognitive decline, making them attractive targets for therapeutic intervention. VEGF-C and VEGFR3 signaling pathways control mLV development and maintenance, and pharmacological enhancement of this signaling can potentially restore drainage capacity in neurodegenerative conditions.

VEGF-C prophylaxis favors lymphatic drainage and improves neurological outcomes after ischemic stroke through enhanced CSF drainage to deep cervical lymph nodes [57]. Age-related changes in meningeal lymphatic function may contribute to the accumulation of neurotoxic proteins and the development of age-related neurodegenerative diseases [58].

## 4. Neuroinflammation and the Tripartite Synapse

### 4.1. Microglial Dysfunction and Synaptic Clearance

Microglial cells serve dual functions as brain immune sentinels and regulators of synaptic plasticity [59]. In neurodegenerative conditions, chronically activated microglia produce neurotoxic factors including tumor necrosis factor-α, nitric oxide, and reactive oxygen species, creating a self-perpetuating inflammatory cycle [59]. This chronic activation is maintained through reactive microgliosis, were neuronal damage signals further microglial activation, creating a feed-forward loop of neurodegeneration. Microglia can be categorized into two opposite types: classical (M1) or alternative (M2), though there’s a continuum of different intermediate phenotypes between M1 and M2, and microglia can transit from one phenotype to another. M1 microglia release inflammatory mediators and induce inflammation and neurotoxicity, while M2 microglia release anti-inflammatory mediators and induce anti-inflammatory effects and neuroprotection [60]. The balance between M1 (pro-inflammatory) and M2 (anti-inflammatory) microglial phenotypes is critically important for neurological recovery. In neurodegenerative diseases, activated microglia are excessively shifted toward the M1 or neurotoxic phenotype due to microRNA dysregulation, particularly involving *miR-124* and miR-155 pathways that control neuroinflammatory processes [61]. M1-type microglia release diverse proinflammatory mediators and free radicals that inhibit brain repair and regeneration. Conversely, microglia of the M2 phenotype improve brain repair and regeneration by enhancing phagocytosis, releasing trophic factors, and reducing brain inflammation. Following stimulation with LPS or IFN-γ, M1 microglia express high levels of inducible nitric oxide synthase and pro-inflammatory cytokines/chemokines such as TNF-α, IL-1β, and CC chemokine ligand 2 [62].

### 4.2. Complement-Mediated Synaptic Pruning

The complement system, particularly C1q and C3 components, mediates synaptic pruning through microglial phagocytosis [63]. While essential for normal development, excessive complement activation in neurodegenerative conditions leads to pathological synapse loss [20]. In pathological conditions such as Alzheimer’s disease, virus infection, or radiation-induced injury, excessive complement-mediated synaptic pruning results in excessive elimination of synapses and is associated with cognitive impairment [63]. C1q localizes predominantly to presynaptic terminals, suggesting that complement-mediated pruning is initiated by presynaptic processes [64]. Recent evidence demonstrates that complement-mediated synaptic loss involves local apoptotic-like mechanisms within synapses, indicating that targeted anti-apoptotic therapies could preserve synaptic integrity [64].

Deletion or blockage of C1q, C3, or CR3 [65] in mouse models of Alzheimer’s disease has been shown to protect synapses and prevent cognitive impairments, highlighting the therapeutic potential of complement inhibition strategies [66]. The role of the complement system in synaptic pruning and neurodegeneration presents novel therapeutic opportunities for controlling excessive synaptic elimination [65]. TREM2, a microglial receptor, modulates complement-mediated synaptic pruning by regulating microglial phagocytic capacity and inflammatory responses [67]. The specific mechanism of TREM2 regulation of synaptic clearance involves modulation of microglial activation states and phagocytic function [67].

### 4.3. MicroRNA-Mediated Inflammation Control

MicroRNA-mediated regulation represents a critical control mechanism for neuroinflammation within the neurovascular-glymphatic dysfunction framework. miR-124 functions as a master regulator of microglial quiescence by targeting pro-inflammatory transcripts and promoting anti-inflammatory M2 polarization through the C/EBPα-PU.1 pathway [27,68]. Loss of miR-124 expression in neurodegenerative diseases contributes to sustained M1 microglial activation and chronic neuroinflammation.

Conversely, miR-155 acts as a pro-inflammatory amplifier, enhancing NF-κB signaling and promoting M1 microglial responses [28,29]. Elevated miR-155 levels correlate with disease severity in multiple sclerosis and Alzheimer’s disease, making it an attractive therapeutic target for anti-inflammatory interventions.

Therapeutic modulation of these microRNA pathways offers precision approaches to control neuroinflammation. miR-124 replacement therapy using lipid nanoparticles or viral vectors could restore anti-inflammatory signaling, while miR-155 inhibition through antagomirs or locked nucleic acid inhibitors could reduce pathological inflammation [61,69]. These microRNA-based therapeutics provide targeted approaches to rebalance microglial phenotypes and preserve neurovascular integrity [70].

## 5. Discussion

### 5.1. Inadequacy of Protein-Centric Approaches

Current therapeutic strategies for neurodegenerative disorders have predominantly focused on reducing pathological protein accumulation, particularly amyloid-β and tau in Alzheimer’s disease [11]. The vascular hypothesis of Alzheimer’s disease proposes that vascular risk factors result in dysregulation of the NVU and hypoxia, which may reduce amyloid-β clearance from the brain and increase its production, leading to both parenchymal and vascular accumulation [11]. Several protein-centric approaches have either been associated with inappropriate immune responses triggering inflammation or have failed to improve cognition, highlighting the need for alternative therapeutic targets beyond protein aggregation [11]. The failure of numerous clinical trials targeting amyloid-β underscores the limitations of protein-centric approaches and suggests that therapeutic interventions must address multiple pathophysiological mechanisms simultaneously. A systematic analysis of failed trials reveals that amyloid-targeted therapies consistently fail to improve cognition despite successfully reducing plaque burden, indicating that neurodegeneration has progressed beyond reversible stages by the time of intervention. In contrast, vascular-targeted approaches offer earlier intervention windows, potentially halting disease progression before irreversible neuronal damage occurs. For example, while aducanumab requires 18+ months to show amyloid reduction with questionable cognitive benefit, VEGF-C treatment demonstrates cognitive improvements within weeks in preclinical models through enhanced clearance mechanisms. This temporal advantage suggests that vascular interventions should be prioritized in early disease stages, while protein-centric approaches may require combination with vascular restoration to achieve clinical efficacy in established disease [11].

The neurovascular dysfunction hypothesis provides a framework for understanding how vascular pathology precedes and potentially drives protein aggregation, offering new avenues for early intervention [11]. Understanding the contribution of neurovascular dysfunction with BBB hyperpermeability to neurodegeneration pathophysiology may help identify novel therapeutic and preventative approaches [9]. These approaches must consider the complex interplay between vascular dysfunction and neuroinflammation, where microglial phenotype balance critically influences therapeutic outcomes [71].

### 5.2. Blood–Brain Barrier Permeability as an Overlooked Target

This temporal relationship suggests that vascular-targeted therapies could potentially halt disease progression before irreversible neuronal damage occurs [5]. Blood-based biomarkers are quantitative, non-invasive diagnostic tools that can identify candidate biomarkers for Alzheimer’s disease using the hypothesis that with BBB dysfunction, brain-synthesized proteins can leak into plasma for detection [72]. Pericytes in Alzheimer’s disease are key players in disease pathogenesis, and transplanted neural stem cells have been shown to alleviate Alzheimer’s disease pathology and cognitive decline, partly by replenishing pericytes [73].

### 5.3. Inflammation-Mediated Neurovascular Damage

The majority of vascular transcriptional changes occur in pericytes, with SMAD3 upregulated in Alzheimer’s disease pericytes having the highest number of ligands including VEGFA, which is downregulated in Alzheimer’s disease astrocytes [74]. Microglia-mediated neuroinflammation is considered a double-edged sword, performing both harmful and helpful effects in neurodegenerative diseases [60,75]. Balancing microglia M1/M2 polarization has a promising therapeutic prospect in neurodegenerative diseases [60].

### 5.4. Precision Medicine Approaches to Neurovascular Dysfunction

Biomarker-guided approaches utilizing vascular dysfunction indicators such as CSF PDGFRβ levels could enable early identification of at-risk individuals before classical pathological changes occur [15]. As outlined in Table 2, these findings have informed the development of multiple promising therapeutic targets addressing various aspects of neurovascular and glymphatic dysfunction, each with distinct mechanisms of action and potential clinical applications based on preclinical evidence.

Models of precision medicine for neurodegeneration focus on developing personalized therapeutic strategies based on individual pathophysiological profiles [1]. The integration of multi-modal biomarker approaches including neuroimaging, fluid biomarkers, and genetic profiling could enable personalized therapeutic strategies targeting specific aspects of neurovascular dysfunction [76]. Biomarker discovery in Alzheimer’s and neurodegenerative diseases focuses on identifying novel targets for early intervention and personalized treatment approaches [77].

**Table 2:** Emerging Therapeutic Targets for Neurovascular-Glymphatic Dysfunction.

Therapeutic Target	Mechanism of Action	Preclinical Evidence	Proposed Therapeutic Approach	Potential Benefits	Challenges/Considerations	Key References
PDGF-BB/PDGFRβ Signaling	Maintains pericyte survival and BBB integrity via ERK and PI3K pathways	PDGFRβ ± mice show accelerated BBB breakdown and neurodegeneration; restoration protects against vascular damage	PDGF-BB supplementation; prevention of PDGFRβ shedding; APOE4-targeted interventions	Preserves pericyte coverage; maintains BBB integrity; prevents early vascular damage	Timing critical; systemic effects; optimal dosing unclear	[35,78]
VEGF-C/VEGFR-3 Signaling	Enhances meningeal lymphatic vessel function and promotes lymphangiogenesis for brain waste clearance	VEGF-C administration in AD mice increases mLV diameter, reduces CSF and brain Aβ, restores cognition	Recombinant VEGF-C (intrathecal or systemic); VEGFR-3 agonists; transcranial radiofrequency stimulation	Enhances protein clearance; reduces tau and Aβ accumulation; improves cognitive function	Delivery route optimization; potential angiogenic effects; dose-finding needed	[30,31]
AQP4 Polarization Restoration	Restores proper localization of AQP4 at perivascular astrocytic endfeet to enhance glymphatic flow	Exercise and calmodulin inhibition restore AQP4 polarization and improve Aβ clearance in AD models	High-intensity interval training; aerobic exercise; calmodulin inhibitors (trifluoperazine); pharmacological AQP4 modulators	Enhances glymphatic clearance; reduces protein accumulation; improves waste removal	Exercise compliance; pharmacological specificity; avoiding edema	[79,80,81]
Complement C1q Inhibition	Blocks initiation of classical complement cascade; prevents C1q tagging of synapses for elimination	C1q deletion or neutralizing antibodies protect synapses and improve cognition in AD mouse models	Anti-C1q monoclonal antibodies; C1q inhibitor peptides; selective C1q blockers	Prevents excessive synaptic pruning; preserves cognitive function; reduces neuroinflammation	Balancing physiological vs. pathological complement; immune surveillance concerns	[19,82,83]
Complement C3 Modulation	Prevents C3 cleavage and iC3b-mediated synaptic tagging; blocks complement amplification	C3 deficiency prevents age-related synapse loss and improves LTP in aged mice; protects against AD pathology	C3 inhibitors (compstatin analogs); C3 convertase inhibitors	Reduces synaptic loss; improves cognitive outcomes; maintains neuronal networks	Timing of intervention; systemic complement functions; infection risk	[18,84,85]
CR3 (CD11b/CD18) Blockade	Prevents microglial engulfment of iC3b-tagged synapses	CR3 knockout mice protected from Aβ-induced synapse loss; reduced microglial phagocytosis	CR3 antagonists; CD11b-blocking antibodies; small molecule inhibitors	Preserves synapses; reduces microglial-mediated damage; maintains circuit function	Microglial function preservation; specificity for pathological pruning	[19,20]
C5aR1 (C5a Receptor) Antagonism	Blocks C5a-mediated microglial activation; reduces excessive synaptic pruning	C5aR1 deletion or PMX205 treatment reduces synapse loss and improves cognition in multiple AD models	PMX205 or PMX53 (C5aR1 antagonists); small molecule C5aR1 inhibitors	Reduces synaptic loss; improves behavior; modulates neuroinflammation without blocking upstream complement	Better therapeutic window than C1q/C3 inhibition; preserves beneficial complement functions	[86,87,88]
miR-124 Replacement Therapy	Restores anti-inflammatory signaling; promotes M2 microglial polarization; inhibits inflammatory mediators	miR-124 overexpression reduces neuroinflammation and promotes neuroprotection in injury models	Lipid nanoparticle-encapsulated miR-124; viral vector delivery; synthetic miR-124 mimics	Shifts microglia to anti-inflammatory phenotype; reduces TNF-α; increases IL-10	Delivery to CNS; off-target effects; stability of miRNA therapeutics	[27]
miR-155 Inhibition	Reduces pro-inflammatory signaling; decreases NF-κB activation; attenuates M1 microglial responses	miR-155 deletion improves outcomes in spinal cord injury and reduces neuroinflammation in MS models	AntagomiR-155; locked nucleic acid (LNA) anti-miR-155; GapmeR inhibitors	Reduces neuroinflammation; improves functional recovery; modulates TLR signaling	Delivery challenges; dosing optimization; potential immune effects	[29,89]
Meningeal Lymphatic Enhancement	Physical or pharmacological enhancement of mLV structure and function	Exercise enhances mLV flow; VEGF-C expands mLV diameter and improves clearance in aged mice	Aerobic exercise protocols; VEGF-C administration; minimally invasive mLV stimulation	Enhances brain-to-cervical lymph node drainage; improves clearance of proteins and immune cells	Age-related mLV degeneration; non-invasive enhancement methods needed	[30,90]
TREM2 Modulation	Regulates microglial phagocytic capacity and metabolic state; modulates complement-mediated pruning	TREM2 deficiency alters microglial response to plaques; affects synaptic engulfment	TREM2 agonistic antibodies; TREM2 activity enhancers (context-dependent)	Modulates microglial function; may enhance beneficial phagocytosis while reducing excessive pruning	Complex role (protective vs. detrimental); stage-dependent effects	[91,92,93]
CD200-CD200R Axis Enhancement	Maintains microglial quiescence; promotes M2 polarization; reduces inflammatory activation	CD200-Fc treatment shifts macrophages/microglia from M1 to M2; reduces pro-inflammatory cytokines	CD200-Fc fusion protein; CD200R agonists	Reduces neuroinflammation; promotes neuroprotective microglial phenotype; decreases oxidative stress	Systemic delivery; CNS penetration; long-term safety	[94]

Abbreviations: PDGF-BB, platelet-derived growth factor-BB; PDGFRβ, platelet-derived growth factor receptor-β; BBB, blood–brain barrier; ERK, extracellular signal-regulated kinase; PI3K, phosphatidylinositol 3-kinase; VEGF-C, vascular endothelial growth factor-C; VEGFR-3, vascular endothelial growth factor receptor-3; mLV, meningeal lymphatic vessels; Aβ, amyloid-β; AD, Alzheimer’s disease; AQP4, aquaporin-4; CR3, complement receptor 3; C5aR1, C5a receptor 1; miR, microRNA; TNF-α, tumor necrosis factor-α; IL-10, interleukin-10; CNS, central nervous system; MS, multiple sclerosis; NF-κB, nuclear factor kappa B; TLR, Toll-like receptor; TREM2, triggering receptor expressed on myeloid cells 2; LTP, long-term potentiation.

### 5.5. Molecular Pathway-Based Therapeutic Targets

Clinical translation of VEGF-C therapies could involve various delivery approaches, including intrathecal administration or systemic delivery with brain-targeting strategies [57].

The prophylactic administration of VEGF-C has shown particular promise, promoting multiple vascular, immune, and neural responses that culminate in protection against neurological damage in acute ischemic stroke models [57]. Therapeutic approaches to central nervous system (CNS) diseases via the meningeal lymphatic system represent a novel frontier in neurodegenerative disease treatment [95]. The meningeal lymphatic drainage provides novel insights into CNS clearance mechanisms and offers new therapeutic targets [56].

Therapeutic targeting of the complement system represents a promising approach for controlling excessive synaptic pruning in neurodegenerative conditions [63]. The complement system in neurodegenerative and neuroinflammatory diseases presents novel therapeutic opportunities for controlling pathological complement activation [96]. Several preclinical complement-targeted therapeutics are in development, focusing on selective inhibition of complement components to preserve beneficial synaptic refinement while preventing pathological synapse loss [63].

MiR-124 and miR-155 serve as therapeutic targets in microglia-mediated neuroinflammation, offering novel approaches for modulating microglial polarization. Therapeutic strategies could involve miR-124 replacement therapy to restore anti-inflammatory signaling or miR-155 inhibition to reduce pro-inflammatory responses. These approaches could be delivered using various platforms including lipid nanoparticles, viral vectors, or conjugated oligonucleotides [61]. Regulation of MicroRNAs in Parkinson’s disease and their potential therapeutic applications demonstrate the broad applicability of microRNA-based therapies [97].

### 5.6. Comparative Therapeutic Efficacy and Intervention Windows

The therapeutic landscape for neurodegeneration requires strategic consideration of intervention timing and target selection [1,76,98]. Vascular-targeted therapies demonstrate optimal efficacy in pre-symptomatic and early symptomatic stages when BBB integrity can still be preserved or restored [5,13,16]. Early detection of BBB breakdown in aging human hippocampus [16] and elevated sPDGFRβ levels in pre-symptomatic individuals [12,14] provide critical windows for vascular intervention before irreversible damage occurs [5,10].

Protein-centric approaches, while potentially beneficial for established disease, show limited efficacy when initiated after significant neuronal loss has occurred [11]. The vascular hypothesis of Alzheimer’s disease proposes that addressing neurovascular unit dysfunction earlier in the disease cascade may be more effective than targeting downstream protein aggregation [11]. Failed amyloid-β trials underscore this temporal limitation, as cognitive benefits remain elusive even when plaque burden is successfully reduced [11].

Cost-effectiveness analyses suggest that early vascular interventions, despite higher upfront screening costs, may provide superior long-term outcomes compared to late-stage protein clearance strategies [76,99]. Biomarker-guided precision medicine approaches utilizing vascular dysfunction indicators such as CSF PDGFRβ levels [12,14,15], albumin quotient measurements [13,16], and complement activation markers [17,18,19] could enable early identification of at-risk individuals before classical pathological changes occur [76,77].

## 6. Future Directions and Research Priorities

### 6.1. Current and Planned Clinical Trials

Several clinical trials are advancing neurovascular-glymphatic therapeutic concepts toward human application. ANX005, a humanized anti-C1q antibody, is currently in Phase II trials for autoimmune diseases with planned expansion to neurodegenerative conditions [83]. VEGF-C enhancement strategies are entering early-phase human studies, building on promising preclinical stroke results [57]. Exercise intervention trials specifically targeting glymphatic function through AQP4 modulation are being developed by multiple research groups. Additionally, transcranial radiofrequency treatments that enhance VEGF expression and improve protein clearance have shown initial promise in small Alzheimer’s disease cohorts [40]. These ongoing efforts represent the first wave of clinical translation for neurovascular-glymphatic dysfunction concepts, with results expected within the next 3–5 years.

### 6.2. Neurovascular Unit-Targeted Drug Delivery

Successful translation of neurovascular-glymphatic therapeutics requires sophisticated delivery strategies that overcome blood–brain barrier limitations [100]. Focused ultrasound combined with microbubbles offers a non-invasive approach for transient BBB opening, enabling targeted delivery of large molecules like VEGF-C and complement inhibitors to specific brain regions [41,101]. Lipid nanoparticle systems, successfully employed for mRNA vaccines, show promise for delivering microRNA therapeutics (*miR-124* mimics or *miR-155* inhibitors) with minimal systemic toxicity [61,89].

Viral vector strategies, particularly adeno-associated virus serotypes with CNS tropism, provide sustained expression of therapeutic proteins but require careful safety monitoring for immunogenic responses [61]. Intranasal delivery represents an attractive non-invasive route for small molecule complement inhibitors, bypassing the BBB through olfactory pathways [95]. Intrathecal administration, while more invasive, offers direct CSF access for biologics targeting glymphatic enhancement [30,31,57]. Each delivery modality presents unique risk-benefit profiles that must be carefully matched to specific therapeutic targets and patient populations [100,102].

### 6.3. Combination Therapy Approaches

The complex nature of neurovascular dysfunction, glymphatic impairment, and neuroinflammation necessitates combination therapeutic strategies, though significant feasibility challenges remain [103]. While preclinical evidence suggests potential synergies, clinical translation faces substantial obstacles including drug–drug interactions, overlapping toxicities, and complex dosing optimization requirements.

Potential combinations include VEGF-C enhancement with complement modulation, though this approach raises concerns about excessive angiogenesis combined with compromised immune surveillance. AQP4 activation with microRNA-based anti-inflammatory therapy requires sophisticated delivery systems and carries risks of off-target gene silencing and unpredictable inflammatory rebound effects [104]. Pericyte protection strategies combined with glymphatic enhancement may produce conflicting vascular effects, as BBB stabilization could paradoxically impede therapeutic drug penetration.

Critical safety considerations include the long-term consequences of complement system inhibition, which may increase infection susceptibility and cancer risk over decades of treatment. VEGF pathway modulation has established associations with cardiovascular complications and hemorrhagic events, particularly concerning aging populations with pre-existing cerebrovascular disease. MicroRNA therapeutics face delivery challenges, immune activation risks, and unknown long-term genomic effects.

The microbiota–gut–brain axis [104] and gut–brain vascular axis [105] represent additional therapeutic opportunities, though mechanistic understanding remains limited and individual microbiome variability complicates standardized interventions. Rational combination design requires extensive phase I safety studies, pharmacokinetic modeling, and biomarker-guided dose optimization considering the temporal sequence of pathophysiological events [106].

Current limitations include lack of predictive biomarkers for treatment response, absence of standardized outcome measures, and insufficient understanding of optimal therapeutic windows. Long-term efficacy and safety profiles for these novel targets remain largely unknown, necessitating cautious clinical development with extensive safety monitoring and adaptive trial designs.

### 6.4. Translational Challenges and Biomarker Development

Translation to clinical applications faces challenges including development of appropriate biomarkers, establishing relevant trial endpoints, and addressing species differences in neurovascular anatomy [99]. The Global Neurodegeneration Proteomics Consortium represents an important initiative for addressing these challenges [99]. Future clinical trials should incorporate adaptive approaches allowing biomarker-guided dose optimization and patient stratification [98]. Advanced biomarkers for BBB dysfunction [15,72] are critical for precision medicine, with biofluid markers for Alzheimer’s disease focusing on vascular and inflammatory indicators [107]. MiRNA neuroinflammatory biomarkers offer novel approaches to monitoring therapeutic responses [108], while reference ranges for CSF PDGFRβ provide clinical assessment standards for pericyte dysfunction [109]. Associations between CSF PDGFRβ, aging, BBB dysfunction, and neuroinflammation provide mechanistic insights into vascular contributions to neurodegeneration [110].

## 7. Conclusions

Neurovascular dysfunction and glymphatic impairment constitute foundational yet underappreciated mechanisms driving the pathogenesis of neurodegenerative diseases. Therapeutic approaches that exclusively target protein aggregation have not sufficiently addressed the complex cascade of vascular and clearance deficits that initiate and perpetuate neuronal injury. This review highlights the critical importance of maintaining NVU integrity, preserving pericyte function, restoring AQP4 polarization, and enhancing meningeal lymphatic drainage as integral strategies to interrupt neurodegenerative progression. Furthermore, modulation of microglial inflammatory phenotypes and complement-mediated synaptic pruning offers additional avenues to mitigate neuroinflammation and synaptic loss. The integration of advanced biomarkers reflecting vascular and glymphatic dysfunction with precision medicine approaches promises to refine early diagnosis and enable tailored interventions. Fostering research and clinical translation targeting these interconnected vascular inflammatory pathways holds substantial potential to transform therapeutic paradigms and improve outcomes for patients with Alzheimer’s disease and related neurodegenerative disorders.

## Figures and Tables

**Table 1:** Key Biomarkers of Neurovascular and Glymphatic Dysfunction in Neurodegeneration.

Biomarker	Source/Location	Pathophysiological Role	Clinical Significance	Detection Method	Key References
sPDGFRβ	CSF, released from injured pericytes	Indicates pericyte injury and BBB breakdown; correlates with neuroinflammation	Elevated in early-stage neurodegenerative disorders; correlates with cognitive decline and BBB dysfunction (QAlb)	ELISA, MSD electrochemiluminescence	[12,13,14,15]
CSF/Plasma Albumin Ratio (QAlb)	CSF and plasma	Reflects BBB permeability; increased ratio indicates BBB breakdown	Correlates with age, pericyte damage, and neuroinflammation; elevated in MCI and AD	Nephelometry, ELISA	[12,13,16]
C1q	Brain tissue, synapses (microglia-derived)	Tags synapses for complement-mediated elimination; initiates classical complement cascade	Increased and localized to synapses before plaque deposition in AD; associated with early synapse loss	Immunohistochemistry, Western blot	[17,18,19]
C3/iC3b	Brain tissue, synapses (astrocyte and microglia-derived)	Opsonizes synapses for microglial phagocytosis via CR3 receptor	Elevated in vulnerable brain regions; C3 deficiency protects against age-related synapse loss	Immunohistochemistry, flow cytometry	[18,19,20]
AQP4 Polarization Index	Astrocytic perivascular endfeet	Maintains glymphatic fluid flow; loss of polarization impairs waste clearance	Depolarization correlates with disease progression and impaired Aβ clearance	Immunofluorescence microscopy	[21,22,23]
CSF YKL-40	CSF (astrocyte activation marker)	Indicates astrocytic activation and neuroinflammation	Elevated in AD and correlates with BBB dysfunction and PDGFRβ	ELISA	[24,25]
CSF GFAP	CSF (astrocyte marker)	Reflects astrocytic reactivity and glial activation	Increased with age and neuroinflammation; associated with BBB dysfunction	ELISA, Simoa	[26]
miR-124	Plasma, CSF, brain tissue	Anti-inflammatory microRNA; maintains microglial quiescence	Downregulated in neurodegeneration; loss promotes M1 microglial polarization	qRT-PCR, sequencing	[27]
miR-155	Plasma, CSF, brain tissue	Pro-inflammatory microRNA; promotes neuroinflammation	Upregulated in MS and AD; correlates with disease severity	qRT-PCR, sequencing	[28,29]
VEGF-C	CSF, brain tissue	Regulates meningeal lymphatic vessel function and lymphangiogenesis	Reduced levels associated with impaired brain clearance; therapeutic target	ELISA, Western blot	[30,31]
CSF Fibrinogen	CSF (blood-derived)	BBB leakage marker; promotes neuroinflammation	Elevated in AD; correlates with pericyte loss and reduced oxygenation	ELISA, immunohistochemistry	[12]

Abbreviations: sPDGFRβ, soluble platelet-derived growth factor receptor-β; CSF, cerebrospinal fluid; BBB, blood–brain barrier; QAlb, albumin quotient; MCI, mild cognitive impairment; AD, Alzheimer’s disease; AQP4, aquaporin-4; Aβ, amyloid-β; GFAP, glial fibrillary acidic protein; MS, multiple sclerosis; VEGF-C, vascular endothelial growth factor-C; MSD, Meso Scale Discovery.

## Data Availability

No new data were created or analyzed in this study. Data sharing is not applicable to this article.

## Abbreviations

Aβ	Amyloid-β
Akt	Protein Kinase B
APOE4	Apolipoprotein Epsilon 4
AQP4	Aquaporin-4
ARG-1	Arginase 1
BBB	Blood–Brain Barrier
C/EBPα	CCAAT/Enhancer-Binding Protein alpha
C1q	Complement component 1q
C3	Complement component 3
C5aR1	C5a Receptor 1
CD200-CD200R	CD200-CD200 Receptor
CR3	Complement Receptor 3
CREB1	cAMP Response Element Binding Protein 1
CSF	Cerebrospinal Fluid
ERK	Extracellular signal-Regulated Kinase
GFAP	Glial Fibrillary Acidic Protein
IL-10	Interleukin 10
IL-1β	Interleukin 1 beta
LPS	Lipopolysaccharide
MAPK	Mitogen-Activated Protein Kinase
miR-124	microRNA 124
miR-155	microRNA 155
NF-κB	Nuclear Factor kappa B
PDGF-BB	Platelet-Derived Growth Factor-BB
PDGFRβ	Platelet-Derived Growth Factor Receptor-β
PI3K	Phosphatidylinositol 3-Kinase
PU.1	PU.1 (also known as SPI1)
Qalb	CSF/Plasma Albumin Ratio
Ser276	Serine at position 276
sPDGFRβ	soluble Platelet-Derived Growth Factor Receptor-β
TLR	Toll-Like Receptor
TNF-α	Tumor Necrosis Factor-α
TREM2	Triggering Receptor Expressed on Myeloid cells 2
VEGF	Vascular Endothelial Growth Factor
